# Advancements in Predictive Microbiology: Integrating New Technologies for Efficient Food Safety Models

**DOI:** 10.1155/2024/6612162

**Published:** 2024-05-17

**Authors:** Oluseyi Rotimi Taiwo, Helen Onyeaka, Elijah K. Oladipo, Julius Kola Oloke, Deborah C. Chukwugozie

**Affiliations:** ^1^Genomics Unit, Helix Biogen Institute, Ogbomosho, Oyo, Nigeria; ^2^School of Chemical Engineering, University of Birmingham, Edgbaston B15 2TT, Birmingham, UK; ^3^Department of Microbiology, Laboratory of Molecular Biology, Immunology and Bioinformatics, Adeleke University, Ede, Osun, Nigeria; ^4^Department of Natural Science, Microbiology Unit, Precious Cornerstone University, Ibadan, Oyo, Nigeria; ^5^Department of Microbiology, Federal University Otuoke, Otuoke, Bayelsa, Nigeria

## Abstract

Predictive microbiology is a rapidly evolving field that has gained significant interest over the years due to its diverse application in food safety. Predictive models are widely used in food microbiology to estimate the growth of microorganisms in food products. These models represent the dynamic interactions between intrinsic and extrinsic food factors as mathematical equations and then apply these data to predict shelf life, spoilage, and microbial risk assessment. Due to their ability to predict the microbial risk, these tools are also integrated into hazard analysis critical control point (HACCP) protocols. However, like most new technologies, several limitations have been linked to their use. Predictive models have been found incapable of modeling the intricate microbial interactions in food colonized by different bacteria populations under dynamic environmental conditions. To address this issue, researchers are integrating several new technologies into predictive models to improve efficiency and accuracy. Increasingly, newer technologies such as whole genome sequencing (WGS), metagenomics, artificial intelligence, and machine learning are being rapidly adopted into newer-generation models. This has facilitated the development of devices based on robotics, the Internet of Things, and time-temperature indicators that are being incorporated into food processing both domestically and industrially globally. This study reviewed current research on predictive models, limitations, challenges, and newer technologies being integrated into developing more efficient models. Machine learning algorithms commonly employed in predictive modeling are discussed with emphasis on their application in research and industry and their advantages over traditional models.

## 1. Introduction

Food safety effectively refers to all measures to guarantee that only food suitable for human consumption is passed on to the human population. Generally, this includes strategies, policies, and legislation to protect food from microbial, chemical, physical, and allergenic contamination during food production, distribution, and consumption [1]. Globally, food pollutants such as heavy metals, mycotoxins, molds, antibiotics, and pesticide residues are becoming increasingly prevalent [2]. The burden of food-borne diseases remains significant with an estimate 600 million illnesses and 420,000 deaths annually [3]. Moreover, the landscape of food safety is evolving due to changing dietary patterns, the industrialization of food production, and the effects of climate change. These factors present new challenges in combating food-borne infections, leading to a rapid increase in cases of foodborne diseases that have adverse effects on public health and the global economy [4].

Moreover, the landscape of food safety is evolving due to changing dietary patterns, the industrialization of food production, and the effects of climate change. These factors present new challenges in combating foodborne infections, leading to a rapid increase in infectious diseases that have adverse effects on public health and the global economy [4].

Globally, food safety measures have evolved significantly to protect consumers on a large scale. In the 19th century, the recognition of the germ theory of disease prompted a shift toward prioritizing sanitation and animal health, leading to the establishment of enhanced safety standards for dairy products. These advancements quickly permeated other sectors of the food industry [5]. The rise of industrialization and mass food processing industries underscored the importance of public health measures aimed at guaranteeing the safety of food consumed by the public. Today, rigorous screening protocols are in place to mitigate microbial hazards (such as pathogenic microbes, metabolites, and mycotoxins), chemical hazards (including heavy metals, pesticides, and carcinogens), as well as allergens and physical hazards like sharp objects [6–9]. Biological hazards pose the most significant threat among these, often leading to devastating outbreaks [10]. According to a review by Lee and Yoon [11], norovirus is associated with the highest number of foodborne morbidities per year globally. It is followed by *Campylobacter*, *Salmonella*, and *Listeria monocytogenes.* Also, a WHO report showed that foodborne bacteria illnesses were seen to have more prevalence than viral and parasitic diseases [12].

Historically, classical food safety measures employed a hazard-based approach relying heavily on a routine inspection of processing facilities and end-product sampling [13, 14]. However, these techniques are highly fallible due to their subjective nature. Hazard-based meat inspection techniques, for example, are based on physical inspection of animal carcasses by incision and palpation to observe for signs of internal organ pathology and lymphadenopathy, which can indicate foodborne pathogens [15, 16]. However, this method may not be effective in detecting diseases such as colibacillosis, campylobacteriosis, or prion-related diseases (e.g., Bovine spongiform encephalopathy) or early infection [17, 18]. Furthermore, invasive practices such as palpation and incision of carcasses are currently being discouraged in most developing countries in favor of a risk-based approach due to their potential to cause cross-contamination of foodborne pathogens [19].

Another hazard-based approach to food safety is the end testing of food products [20]. However, this presents several challenges; first, it is a reactive rather than a proactive approach; in other words, this method is used to detect microbial contamination rather than prevent it from occurring in the first place. Second, negative results (indicating no microbial contamination) do not guarantee that the product is entirely free from contamination [21]. Moreover, this method, which is predominantly carried out through microbial culture techniques based has limited sensitivity and is limited by the microbial load present; consequently, the absence of a particular microbe during testing does not necessarily mean they were never present or will not appear later. Due to their limited sensitivity, culture-based methods of end testing are also prone to false positives and false negatives and are currently being replaced, or in some cases, augmented by molecular detection methods (such as whole genome sequencing) which are more accurate [22]. Hazard-based end testing of food is also labor intensive and costly due to the expenses associated with the technique and the potential losses incurred upon detecting microbial contamination [23].

Moreover, relying solely on a hazard-based approach can be impractical and unsustainable. Strict enforcement of this approach may prohibit essential and nutritious foods if they contain naturally occurring toxins, such as genotoxic hydrazines in mushrooms, glycoalkaloids in tomatoes and potatoes, and hydrocyanic acid in cassava. Furthermore, traditional food processing methods like frying potatoes have been linked to producing carcinogens such as acrylamide. Banning these food items and cooking techniques outright may be unrealistic, particularly in developing countries. Such a ban could significantly disrupt food availability and consumption [24].

As part of measures towards overcoming these challenges with hazard-based approaches, the World Trade Organization (WTO) and the Codex Alimentarius Commission (CAC), in collaboration with different public health bodies and national governments, came up with a regulatory risk-based framework on food hygiene, processing, and practices in the food industry. The Codex Alimentarius Commission was founded in 1963 as an intergovernmental body under the Food and Agriculture Organization United Nations and the World Health [25]. During the 19^th^ and 20^th^ sessions of the CAC in 1991 and 1993, the decision was taken to base food safety decisions on risk assessment; the committee also accepted a recommendation to harmonize standard-setting methodologies. The drafting of the Codex Alimentarius document on “Guidelines for the application of the Hazard Analysis Critical Control Point (HACCP) system” (Codex, 1993) and the subsequent edition, “Hazard Analysis and Critical Control Point System and Guidelines for its Application” [26] played a key role in the adoption of HACCP protocols in food safety globally [27, 28].

Towards the end of protecting consumers, a risk analysis framework prescribing regulations such as the Appropriate Level of Protection (ALOP) and the Food Safety Objective (FSO) was developed. The ALOP, also known as ALR (Acceptable Level of Risk), is a concept used in risk analysis to establish the appropriate level of protection against hazards in food production and ensure the safety of food for human consumption. It is described as “the level of protection deemed appropriate by the member establishing a sanitary or phytosanitary measure to protect the human, animal, and plant life or health within its territory” [29]. The determination of the ALOP or ALR is based on a combination of scientific knowledge and expert judgment, taking into account factors such as the severity and likelihood of harm, the availability and effectiveness of risk mitigation measures, societal values, and expectations, as well as epidemiologic surveillance data on the occurrence and distribution of foodborne illnesses.

The Food Safety Objective (FSO) is a metric that describes the maximal threshold of a microbial hazard in a food at the point of consumption; it was designed to translate the ALOP into practical measures for stakeholders in food manufacturing and distribution [1, 30]. Maximal threshold hazard levels at other points on the food processing chain are called performance objectives (POs) [31]. Other regulatory standards developed in line with the risk-based safety approaches include GAP (good agricultural production practices), GHP (good hygiene practices), GMP (good manufacturing practices), and HACCP [32].

Adopting risk-based approaches in food safety has shifted objectives towards preventing hazards rather than relying solely on inspection-based methods [33]. This shift acknowledges the importance of proactively identifying and mitigating risks to food safety. While these approaches have contributed to improved food safety globally, there is a growing recognition of the need for increased efficiency, precision, and accuracy in food safety practices [34]. As a result, researchers are turning to mathematical microbial modeling to complement existing methods [35]. Predictive microbiology, in particular, has emerged as a valuable tool.

Microbial predictive modeling involves using mathematical models to predict the growth and behavior of microorganisms in food products under different environmental conditions [36]. This field of study is focused on developing models that can help food manufacturers and regulators understand how microorganisms respond to changes in factors such as temperature, pH, water activity, and other environmental conditions. The objective is to utilize this knowledge in forecasting the potential growth of harmful foodborne microorganisms and to predict the potential growth of harmful microorganisms that can cause foodborne illness [37, 38]. Predictive microbiology is established on the notion that the behavior of microorganisms, however complex, can be simulated and effectively reproduced in nonbiological models by applying mathematical and computational principles [35]. By utilizing mathematical models, predictive microbiology enables the identification of microorganisms of interest and provides insights into microbial ecology, sources of contamination, and potential contamination points. Predictive modeling plays a crucial role in ensuring food safety by allowing food manufacturers and regulatory agencies to anticipate and control the growth and behavior of microorganisms in food products. The goal of predictive microbiology is to provide tools that can be used to design food processing and preservation strategies that can help reduce the risk of foodborne illness and improve the safety and quality of food products.

The application of predictive microbiology has found its way into diverse areas of food microbiology, such as analysis of food formulation processes and their effect on shelf life, evaluation of processing operations, and hazard analysis critical control point (HACCP).

Currently, the advancement of modeling techniques in food safety is heavily influenced by the integration of artificial intelligence (AI) and other cutting-edge technologies, such as whole genome sequencing [39, 40]. Machine learning algorithms such as random forest models and support vector machines (SVM) are becoming integral components of these evolving models [41, 42]. Whole genome sequencing (WGS) technologies, alongside genomics and other omics-based methods, generate vast amounts of high-throughput data. These data serve as invaluable resources for training models using various AI algorithms [43]. By harnessing the power of AI, researchers can effectively analyze and interpret complex datasets to derive meaningful insights. These sophisticated modeling tools find applications across diverse domains within food safety, notably in risk assessment and management.

The use of predictive models in food safety complements risk-based safety approaches; it also has several advantages over traditional microbiological testing, including the following.

Enhanced speed and accuracy: predictive models can quickly and accurately predict the growth and behavior of microorganisms in food products, allowing for faster and more accurate risk assessments and decision-making.

Improved risk assessment: predictive models can provide more detailed and accurate risk assessments of food products, helping to identify potential hazards and develop effective control measures.

Cost savings: using predictive models can reduce the need for expensive and time-consuming microbiological testing, saving both time and money.

Optimization of food processing and preservation methods: predictive models can optimize food processing and preservation methods, ensuring that food products are safe, high quality, and have a longer shelf life.

This review aims to provide a comprehensive overview of the field of predictive microbiology and its applications in improving food safety. It also addresses current limitations and challenges in this field and explores emerging trends and approaches, including the use of new technologies such as genomics and metagenomics.

### 1.1. Factors Affecting Microbial Growth in Food

During the preharvest stage, food crops are vulnerable to contamination in the field [44]. This can happen through contact with waterborne pathogens during irrigation, exposure to soil-borne pathogens present in manure, or contact with wildlife such as migratory birds [45]. The harvest stage is another critical point where contamination can occur. Food crops are susceptible to contamination as they are picked by workers, who may introduce pathogens through improper handling practices [46]. Postharvest processes, including transportation, processing, distribution, and preparation, also present opportunities for microbial contamination. Food can come into contact with soil, air, and waterborne microorganisms during these stages, increasing the risk of contamination [45]. In animal-based foods like beef, microbial contamination can originate from high microbial loads present in the cattle gut [47]. During carcass preparation, there is a risk of transferring these pathogens to skeletal muscle, further emphasizing the importance of stringent hygiene practices throughout the food processing chain [48].

Most foods provide an ideal nutrition source for microorganisms [49]. Generally, factors influencing microbial growth in food are classified into intrinsic and extrinsic factors. Intrinsic factors are physicochemical characteristics inherent to the food, such as composition, pH, water activity, oxidation-reduction potential, and biological structures. On the other hand, extrinsic factors encompass environmental conditions surrounding the food, including temperature, relative humidity, gaseous environments, and competitor microorganisms [49].

Intrinsic factors play a pivotal role in microbial growth dynamics. Foods of plant or animal origin possess biological components that act as barriers against microbial invasion and colonization. Examples include the tough testa and shells of certain seeds and nuts and the thin membrane found in eggs. In addition, readily available nutrient sources in food significantly influence microbial proliferation. Bacteria typically require simple sugars and free amino acids for metabolism, favoring their growth in nutrient-rich environments [50]. Conversely, microbial growth can be hindered by natural antimicrobial substances such as bacteriocins, allicins, and eugenol [51]. Extremes of pH, such as highly alkaline or acidic conditions, also deter bacterial growth as most bacteria thrive within a narrow pH range compared to molds and yeasts [52].

Considering the water activity requirements of microbes, it is notable that most bacteria are halophiles (salt loving). Gram-negative bacteria typically exhibit a greater affinity for water than Gram-positive bacteria, molds, and yeasts. Consequently, fast-growing bacteria are often the primary spoilers of fresh foods with high water content [50].

Moreover, additional factors influencing bacterial growth in food encompass implicit factors related to the microbes, including interactions between different microbial species colonizing the food. These interactions may involve differing nutrient utilization, stress thresholds, and the production of chemicals that modulate the growth of other microorganisms. Processing factors, such as heating, cooling, and drying treatments, also significantly affect food composition and microbial ecology. Ultimately, a complex interplay between all the factors above dictates microbial growth dynamics in food systems [53].

## 2. Principles of Predictive Microbiology

The basic principle of predictive microbiology is anchored on the knowledge that microbial growth and survival can be simulated in mathematical models.

The effectiveness of mathematical growth models is affected by different criteria. More excellent resolution and predictive relevance can be achieved by reducing the number of parameters under analysis. Furthermore, a mechanistic approach assessing few meaningful parameters allows for more phases of microbial growth to be covered and consequently increases the model's accuracy [54].

### 2.1. Microbial Growth Models

The growth curve of most foodborne bacteria typically exhibits the following four distinct phases: an initial lag phase characterized by minimal or no detectable growth, followed by an exponential (log) phase marked by rapid cell division, then a plateau phase where growth stabilizes, and finally, a mortality phase where conditions become unfavorable for further growth [55].

Microbial models are classified based on a number of different criteria. They can be grouped into kinetic and probabilistic models based on the expected microbial response; empirical and mechanistic models based on the method of analysis; and primary, secondary, and tertiary models based on the dependent variable assessed [29, 56, 57].

#### 2.1.1. Primary Models

Primary models assess bacterial response over time to specific conditions. Typically, these models aim to describe microbial growth using as few parameters as possible [58]. Primary models may be empirical, rate growth models, inactivation or survival models, or a combination thereof. Examples include the Gompertz and Buchanan models [59]. These models evaluate a discrete number of intuitive parameters, such as the relative growth rate, initial population size, and asymptotic population size. However, they may be unable to assess all microbial growth phases or account for variables such as prolonged lag phases [54].

The Gompertz equation is commonly written mathematically as

*L* = *A* + *C*exp {−exp [*B*(*T* ∼ MI]} [60].

It describes an asymmetric sigmoidal curve.

*L* = log+ of bacteria count in colony forming units(cfu); *t* = time in hours; *A* ∼ asymptotic log count with a decrease in time indefinitely; *C* is asymptotic log count with an indefinite increase in time; *M* is the time at the maximal growth rate; and *B* is the relative growth rate at time *M*.

This model has been used by the Food MicroModel consortium and the Pathogen Modelling Program (PMP) group in the UK and the US, respectively [61]. The model, however, is limited in its ability to describe the exponential phase. It also overestimates parameter values [62–64].

The Buchanan model:

(1) $$\begin{array}{c} \log\left(N\right)=\left\{\begin{array}{cc} \log\left(N\right) & \left(t\leq r\right),\\ \log\left(N_{0}\right)+\mu_{\max} & \left(t-r\right)\;\left(r<t<t_{m}\right),\\ \log\left(N\;\max\right) & \left(t\geq t_{m}\right). \end{array}\right. \end{array}$$

(2) $$\begin{array}{c} \tau =\tau_{i}+t_{m}\;i=1,2,\cdots ,N, \end{array}$$

(3) $$\begin{array}{c} \mu_{\max}=\frac{\log\left(N_{\max}\right)-\;\log\left(N_{0}\right)}{t_{m}-\tau }. \end{array}$$

Equations (1)–(3) express assumptions by the model in simulating growth. The Buchanan model assumes that there is zero growth during the lag phase, and growth during the exponential phase increases linearly with time and specific growth in the plateau phase is zero.

The Buchanan model was developed based on the following three assumptions: (1) during the lag phase, the specific growth rate is zero; (2) during the exponential phase, the logarithm of the bacterial population increases linearly with time; and (3) during the stationary phase, the specific growth rate is zero [65]. It is also regarded as a very simple model, making it very popular for microbiological modeling [65].

#### 2.1.2. Secondary Models

Secondary models analyze the factors influencing the kinetic parameters identified by primary models. They establish the relationship between primary model parameters and intrinsic and extrinsic factors such as temperature and pH [66]. While primary models focus on estimating changes in microbial population over time and observing specific responses such as growth rate and lag phase, secondary models assess the impact of intrinsic and extrinsic factors of the food on these responses [29]. Several secondary models have been developed to evaluate the lag phase and growth rate concerning one or more environmental or physicochemical factors [58, 67]. Examples of secondary models include the response surface (RS) models and Arrhenius models [68]. A response surface model in predictive microbiology is a mathematical model used to describe the relationship between multiple independent variables (factors) and a response variable, typically microbial growth or survival, in a complex environment [69]. Response surface models (RSMs) are crucial in predictive microbiology. They help us understand how different factors such as temperature, pH, and humidity affect microbial growth or survival [70, 71]. These models show how these variables work together, impacting the microorganisms' environment. RSM works by mapping out a multidimensional space to predict outcomes based on various factors. It involves conducting experiments where these factors are varied systematically and then using math and statistics to build a model representing the response across all the experiments [72]. This model's surface guides us to find the best conditions for controlling microbial growth or death. In predictive microbiology, RSM is used to fine-tune food storage and safety. It helps find the right mix of conditions, such as temperature and moisture, to prevent harmful microbes from growing in food. For example, RSM can help determine the exact temperature and humidity levels needed to prevent pathogen growth in food products [70].

#### 2.1.3. Tertiary Models

Tertiary models are algorithm-run software packages combining primary and secondary models with a Graphic User Interface (GUI), making them userfriendly for amateur modelers. They are primarily used in the food industry and research to consolidate findings from primary and secondary models. The Unified Growth Prediction Model (UGPM) software is an example of a tertiary model based on Baranyi Roberts's primary and temperature-dependent secondary models [73].

### 2.2. Empirical and Mechanistic Models

#### 2.2.1. Empirical Models

Empirical models are pragmatic, rigid models in which relationships between different parameters are put forth as mathematical equations, usually in first or second-degree polynomial mathematical relationships [58, 68]. In an empirical model, predictions are made without considering variables such as physicochemical parameters capable of influencing the outcomes of predictions. An example is the quadratic response surface used by Gibson et al. [74].

#### 2.2.2. Mechanistic or Deterministic Models

Mechanistic or deterministic models are established on theories and allow interpretation of the response in terms of known phenomena and processes. These models typically contain fewer parameters, fit the data better, and describe the response better. They are also known to extrapolate more effectively and are preferable to empirical models [66].

### 2.3. Kinetic and Probabilistic Models

#### 2.3.1. Kinetic Models

Kinetic models determine the expected response rates (growth or death). They are used to predict concentration levels associated with a certain level of microbial strain and thus characterize risk (infection or intoxication-associated risk) [58]. Examples are the Gompertz and square root models, which describe rates of response, such as lag time, specific growth rate, and maximum population density, or inactivation/survival models that describe destruction or survival over time [75–78].

#### 2.3.2. Probability Models

Probability models, on the contrary, are associated only with the probability of growth or toxin production and do not predict the rate at which this occurs [79, 80]. They are used to show the absolute limit of microbial growth within specific environments and demonstrate stress threshold levels, which may limit growth but ultimately permit it [79, 80].

### 2.4. Applications of Predictive Microbiology in Food Safety

#### 2.4.1. Quality Control of Food Products

Predictive microbiology can be applied to validate the effectiveness of microbial inactivation processes such as drying, heat treatment, and refrigeration. Food processing industries producing yogurt, milk, wine, and sous-vide processed products require strict adherence to specific refrigeration temperature and heat treatment [81–83]. However, these treatment processes are often inadequate or improperly managed, leading to microbial growth. In the dairy industry, for instance, the insufficiency of heat treatments solely as the control measure against fungal spore germination has been highlighted; this is due to the ability of some spore-forming fungal pathogens to survive pasteurization or thrive at low temperatures. Organisms such as *Bacillus sporothermourans* are known to survive the ultrahigh temperatures reached during heat treatments [84, 85]. Several research studies on cold chains have also revealed severe noncompliance with regulations at distribution, retail, and storage with the final consumer. This, therefore, necessitates the need for quality control points along the food chain.

A study by Gougouli et al. [86] demonstrated that microbial growth in yogurt, evidenced by signs like mycelial formation, is significantly influenced by variables such as warehousing duration, storage temperature, and microbial strain. Consequently, predictive models capable of anticipating fungal growth by analyzing these factors are highly valuable in the dairy industry. These models can enhance quality control measures by integrating them into the final testing of yogurt products for fungal growth before market release. In such applications, predictive models aid in determining optimal conditions for the growth of various microbial species in end-product challenge testing.

It is essential to recognize that different microbial contaminants have varying conditions permissive for growth. Therefore, the selection of environmental conditions during end-challenge tests cannot be made arbitrarily without a solid scientific basis, as these can lead to false negative tests and inadvertent marketing of contaminated products. Other predictive models in the dairy industry have modeled the influence of parameters such as temperature, pH, water activity, and inoculum size on the growth of *Listeria monocytogenes* in milk as well as the growth of *Yersinia enterocolitica* in Camembert-type cheese [87].

#### 2.4.2. Risk Assessment and Management

Risk assessment is a systematic, scientific method designed to calculate human risk associated with exposure to foodborne risks [26]. It comprises the following four sequential stages: hazard identification, exposure assessment, hazard characterization, and risk characterization [88, 89]. Data from MRA are heavily utilized in developing policy-making and legislation for the most relevant foodborne pathogens [25].

Conventional methods for risk assessments of food products and additives are deterministic [90]. They are based on the assumption that estimated parameters are constants, while these parameters are variables, and their measurements are uncertain. Consequently, discrepancies between challenge tests and laboratory assays lead to compromised decisions when deterministic approaches are utilized alone in decision-making. Hence, more sensitive methods, such as predictive modeling, are necessary. By applying predictive models in the food industry, microbial hazards can be estimated from production to final consumption [91]. Information from these models can then be utilized in guiding decisions such as determining acceptable levels of microbial exposure and necessary actions to minimize risk to the final consumer [20].

A study was conducted by Pouillot and Lubran. [92] to assess the effectiveness of different established predictive models in quantitative risk assessment of listeriosis from contaminated cold-smoked salmon and the growth of *L. monocytogenes.* In this study, parameters such as the initial log_10_ concentration of *L. monocytogenes* temperature fluctuation in a cooling device and storage duration were monitored using the dose-response model for invasive listeriosis developed by [89].

Pr (illness|*D*) = 1 − exp (−*rD*), and *D* = *C* × 10^*X*end^

With *r* as the probability of illness after ingesting one *L. monocytogenes* (number of organisms), the dose-response *r* was deemed *r* = 1.06 × 10^−12^ (based on data from an FAO report on population susceptibility).

D is the predicted dose of *L. monocytogenes* per serving.

*X*_end_ (log_10_ cfu/g) = concentration *of L. monocytogenes* in the cold-smoked salmon after storage.

The final output of the model was calculated using Monte Carlo simulation as

- $\overset{¯}{R}$ = 1/*n*∑_*i*=*n*_^*n*^(1 −  exp(−*r* × *C* × 10^*Xend*,*i* ^)).

Different models, including the Buchanan model and the Baranyi model, were assessed. The result of the study showed the importance of accurately measuring bacterial growth in determining risk and the significance of selecting an appropriate primary growth model. The study also revealed the effect of different parameters on the estimated risk. Parameters such as the maximum achievable population density and lag phase were seen to be important in risk estimation.

The study also highlighted the gap between predictive microbiology models and current models being used in microbial risk assessment, as well as the difficulty in selecting appropriate models.

MicroHibro is an example of a predictive microbiology modeling software for evaluating spoilage and pathogenic microbes in the food industry. It provides estimates on the exposure level and associated risk. The software utilizes a Predictive Microbiology Model Database (PMDB), incorporating parameters such as growth, inactivation, transfer, and dose-response models. Estimating food microbial risk is done in MicroHibro by describing steps in the food chain using four microbial processes (growth, inactivation, transfer, and partitioning). It then estimates the microbial load and prevalence in the food of interest [93]. Other risk assessment tools that have been developed include the Dairy Products Safety Predictor, Food Spoilage and Safety Predictor, Listeria Meat Model, and FDA-iRISK [94].

#### 2.4.3. HACCP (Hazard Analysis and Critical Control Point) Systems

Hazard Analysis and Critical Control Point (HACCP) is a food safety system designed to identify and prevent potential problems throughout food production, distribution, and consumption.

It employs a systematic approach to identify pathogens in raw materials and processing entry points, implement appropriate methods for their elimination, and detect potential issues with the finished product resulting from improper handling. Predictive food microbiology plays a crucial role in implementing the HACCP concept.

The application of Quantitative Microbial Risk Assessment (QMRA) is essential in hazard analysis by evaluating potential microbial hazards in the food chain and determining critical control points (CCPs) and critical limits (CL). Predictive models can also be developed to evaluate systems established to monitor CCPs and verify the effectiveness of the HACCP system. These models can predict CCPs by determining levels for different parameters that permit microbial growth. In addition, they estimate various levels of microbial behavior to suggest permissible levels and thresholds for critical limits. Hence, integrating HACCPs with predictive models has excellent potential in decision-making [88].

In a study by Cassin et al. [95]; Monte Carlo simulation was used together with data from microbiological analysis in a dose-response analysis to predict the risk of hemolytic uremic syndrome (HUS) from *Escherichia coli* O157: H7 from beef hamburgers. The study modeled the pathogen behavior through processing, handling and final consumption and the determined the risk of the illness in different age groups. The authors then went on to assess the effectiveness of different mitigation strategies and critical control points in storage temperature control, preslaughter screening, and cooking temperature. From their results, the probability of illness was reduced through a strategy aimed at minimizing the bacteria growth during retail storage through accurate temperature monitoring. However, the authors also acknowledged the limitation of uncertainties in hygiene during individual operations during processing, production and handling. The authors were also unable to model certain steps in the production, processing, hamburger distribution, and consumption during the study. For instance, not all bacteria that survive the cooking process would be infective after. Also, the probability of HUS cannot be totally ascribed to dose.

#### 2.4.4. Shelf-Life Determination

Traditional microbiological methods for determining shelf life are often time consuming, requiring substantial bacterial cell growth before overt spoilage reactions become apparent. Alternatively, newer methods may require advanced and expensive equipment. However, knowledge gained from predictive modeling of microbial behavior in food provides a robust foundation for developing devices capable of monitoring food shelf life throughout warehousing, transport, distribution, and retail sales.

Accurate shelf-life prediction models must fulfill several requirements, including identifying spoilage reactions (SRs) such as slime production, color changes, and offensive odors. They must also identify the specific microorganisms (SSOs) responsible for these spoilage reactions and analyze the spoilage domain (SD), which refers to the environmental conditions permitting the growth and function of a particular SSO. Developing and validating a shelf-life prediction model involve experiments demonstrating the spoilage organisms, reactions, and domain. Subsequently, modeling microbial behavior within this domain is crucial for determining the “minimum spoilage level,” i.e., the concentration of SSO required to cause product rejection. Notably, spoilage organisms' metabolic activities, rather than their numbers alone, play a significant role in determining spoilage [96].

Numerous predictive models have been developed for shelf-life studies in the food industry. Examples include models for predicting shelf life in yogurt by Mataragas et al. [97]; minced beef by Limbo et al. [98]; and Nutri cereal baby food by Rasane [99]. In addition, studies have focused on the impact of specific bacteria on spoilage in particular foods, such as *Pseudomonas* on pork and poultry by Bruckner et al. [100] and lactic acid bacteria on cooked ham by Kreyenschmidt [101]. These predictive models enhance food quality and safety by providing insights into the factors influencing shelf life and potential spoilage mechanisms.

### 2.5. Limitations of Predictive Models

Models provide many benefits in the decision-making process. However, it must be kept in mind that they are simplified representations of complex biological processes at best. Therefore, predictions based on model results should be made cautiously, considering previous experiences and other microbial ecological factors for which the models may not fully account. For instance, it is crucial to understand that models can only be extrapolated for values within the experimental ranges to which they were formulated, especially for parameters like temperature or water activity. This limitation arises because models, particularly empirical ones, are derived from fitting observed data and may only partially simulate actual microbial behavior. Research by Fakruddin et al. [68] also reports that specific models may predict faster growth rates compared to actual real-life scenarios. This discrepancy has been attributed to the development of most models using laboratory media, reducing their predictive relevance in the food industry even though they are validated in foods [102]. Practitioners in the food industry have also highlighted the incompatibility of models developed under stable environmental conditions in evaluating real-life products that experience fluctuating conditions of changing temperature, pH, and water activity. Moreover, predictive models cannot consider all the variables that affect food spoilage and microbial growth [103]. Most models evaluate just one or more parameters involved in food spoilage [104].

Researchers have also highlighted several more specific challenges, such as the prediction of the lag phase. The lag phase is a period of adjustment with little or no bacteria multiplication. Modeling this phase of bacterial growth has been challenging due to the variable length and the minimal level of microbial growth [103]. Population kinetics factors, like the lag phase and growth rate, are not easily predicted in rapidly evolving, complex ecosystems such as that of natural food. Modeling the shelf life of fish and seafood has also been difficult due to the scarcity of data on changes in fish microbial response after subjecting them to temperature changes during the lag and/or the exponential phase. Therefore, applicable and accurate models for quantitative microbial risk assessment in seafood are unavailable [105].

Other limitations with the use of predictive models include the inability of models to model intraspecies diversity, the effect of natural food structure complex, as well as the effect of nutrient depletion and buildup of toxic bacteria waste in the medium [106, 107].

#### 2.5.1. The Role of Industry Stakeholders in the Adoption of Predictive Models

Industry stakeholders applying modeling in food safety need to be informed of the predictive limits of models before making inferences. Thus, proposed models must be backed up with evidence showing a track record of valid predictions to aid adoption.

Table 1 outlines the role of AI, whole genome sequencing (WGS), and metagenomics in developing more efficient and accurate predictive models.

## 3. Challenges and Future Directions

Most microbial models are simplistic, primarily describing observable changes in response to environmental dynamics. While these models have proven effective in predicting parameters such as growth or inactivation rates within optimal temperature ranges, their ability to model complex phenomena like lag phases and adaptive responses remains questionable. Consequently, there is a growing need for more versatile and mechanistic next-generation models that integrate cellular information to unravel complex microbial behavior [109, 120].

Newer modeling approaches should prioritize understanding the behavior of individual cells to comprehend the dynamics of entire populations more accurately. Recent studies have emphasized the significance of individual cell heterogeneity as a significant source of variability [121, 122]. Given that food contamination can occur with very low numbers of pathogenic bacteria, understanding the mechanisms of individual cell behavior is crucial for robust microbial risk assessment [123, 124].

To address these challenges, research is underway on stochastic individual cell modeling based on systems biology approaches. These studies have revealed the variation of bacterial cells within a clone and the association between gene expression and phenotypic expression [125].

Next-generation models must also analyze complex interactions such as intraspecies diversity found in spore-forming bacteria and their relevance in risk assessment. This characteristic has not been adequately modeled in current approaches [126].

### 3.1. Integration of Predictive Microbiology with Other Technologies

Advances in whole genome sequencing (WGS) technologies, genomics, and metagenomics are revolutionizing pathogen detection, characterization, and identification in the field of food safety, giving rise to an emerging field known as foodomics [39, 127]. Furthermore, proteomics and metabolomics methods are being applied in detecting bacterial toxins and mycotoxins in foodstuffs, making omics-based tools a vital aspect of risk assessment methods and food safety surveillance in the 21st century. The integration of WGS in surveillance programs has been applied in resolving outbreaks of listeriosis and Salmonellosis that previously would not have been detected [128–130]. By applying WGS technologies in predictive microbiology, researchers have access to details such as serotype, virulence factors, antimicrobial resistance genes, and genetic variations such as single nucleotide polymorphisms (SNPs) [39, 118]. This enables more precise risk assessment that better translates microbial genotype into differing clinical outcomes [131]. For instance, Shiga toxin-producing *Escherichia coli* is associated with different clinical outcomes ranging from diarrhea to hemolytic uremic syndrome (HUS) and other chronic sequelae. Applying WGS technologies in risk assessment makes it possible to link different hazards with particular clinical outcomes accurately [118, 132]. This was carried out in a study by Pielaat et al. [133] in which molecular data from WGS were applied to characterize hazards from *Escherichia coli* O157: H7 [134].

In addition, metagenomics can be used to study the interactions between microorganisms in a community [116]. Microbial communities are complex systems, and one microbe's behavior can influence others' behavior. By using metagenomics to study these interactions, it is possible to develop more sophisticated predictive models that take into account the dynamics of microbial communities.

Another tool with massive potential for integration into current predictive modeling systems is artificial intelligence and machine learning (ML) technology. The process of developing a predictive model generates large datasets that often cannot be analyzed using conventional statistical tools. Artificial intelligence and machine learning tools can be used to analyze large datasets and identify cryptic patterns and correlations. Artificial Intelligence, therefore, makes it possible to develop more sophisticated and accurate models that consider multiple factors that influence microbial growth and behavior. Several scientific reports have been written on the application of AI in this field [40, 43].

Machine learning is a subset of artificial intelligence focused on developing more efficient machines by integrating algorithms that enable them to learn from available datasets. Machine learning algorithms learn over time and are being employed in a holistic approach towards risk prediction due to their ability to consider complex genetic variations and interactions [135]. They allow for the evaluation of individual parameters and their interactions with other variables, which are vital in assessing strain diversity and the differences in observed phenotypic outcomes [136]. Microbial risk assessment incorporating these algorithms can capture changes in genetic constitution with time and consequently aid in faster identification of new strains with novel virulence characteristics [131]. It would also help in resolving limitations associated with predictive models. Machine learning algorithms are more flexible and dynamic than conventional predictive microbiology models as they do not require rigid formulas linking microbial parameters and observed features in food [137]. Algorithms employed in ML can discern complex relationships between input data and, from thence, make predictions [131]. Therefore, they do not rely on additional theories or mechanisms (as in mechanistic or empirical predictive models). AI is trained with large datasets, too many parameters to learn complex relationships, making it possible to predict even without clearly understood mechanisms accurately [137].

Machine learning models have also been shown to be more adept at exploring complex interactions among input features (e.g., growth kinetic parameters, type of pathogen, and sublethal injury rate) with fewer parameters [114].

A study by Lin et al. [114] applying machine learning for predicting the single-cell lag time of *Salmonella enteritidis* after heat and chlorine treatment on single-cell analysis demonstrates the following. Unlike traditional models, which need different sets of parameters to fit the data for varying microbes and processing methods, AI model requires only a set of hyperparameters when evaluating the impact of a sublethal injury on single-cell lag time, making it possible to build a single model capable of assessing single-cell variability among different pathogens to different treatments; an ideal model for all rather than a model per pathogen per treatment process [138].

**Figure 1:**
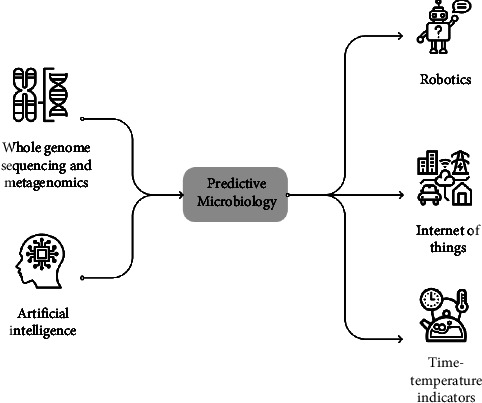
Integration of newer technologies into predictive modeling. WGS and metagenomics help to understand the abundance and diversity of microbial communities present in food samples. Data generated by these tools are then analyzed by artificial intelligence (AI) and machine learning (ML), leading to the development of accurate, sophisticated algorithms that are being utilised in the development of robots, time-temperature indicators, and internet of things (IoTs) devices. Image sources: Arthur Shlain; dDara TH; rukanicon ID; icon scout IN; WiStudio TH.

These tools have also helped automate food quality monitoring systems through technologies such as robots, time-temperature indicators (TTIs), and Internet of Things (IoTs) technologies. Through the integration of predictive models with artificial intelligence technologies, robots are being developed to optimize the handling and storage of products, reducing the risk of microbial contamination [139].  Figure 1 illustrates the integration of newer technologies into predictive modeling. IoT devices can be used to monitor and control the temperature and humidity of food and other products. Time-temperature indicators (TTIs) are devices that monitor the time-temperature history of a product and provide an indication of the degree of microbial growth. By integrating these technologies into current predictive models, it is possible to monitor and maintain the conditions required for the safe storage and distribution of products. Predictive models are developed to ensure that conditions are optimized for food safety, while artificial intelligence technologies help to add human-like awareness and decision-making in product design [140].

### 3.2. Machine Learning Algorithms Commonly Employed in Predictive Modeling

Many different ML algorithms, such as gradient boosting regression tree (GBRT), artificial neural network (ANN), random forest (RF), support vector regression (SVR), and logit boost (LB), are commonly used [114, 141].  Table 2 shows the application of different machine learning algorithms that have been used with varying levels of success.

#### 3.2.1. Random forest

This algorithm derives its name from the approach towards data selection and decision-making. Generally, a random forest algorithm works by integrating multiple decision trees into a single ensemble of models based on the principle that the aggregation of multiple models should perform better than a single one. The algorithm randomly selects input variables and observations from provided data, from which it then generates individual decision trees. The final result, hence, is an aggregation of multiple trees for regression. Random forest algorithms have shown suitability in predictive microbiology due to their accuracy with classifications and regression analysis and suitability in assessing causality between parameters and effects; RF also considers the significance of variables in handling data. It is also rather easy to use and understand due to the small number of parameters to optimize (count of decision trees and independent variables per split) [154]. Random forest (RF) methods, particularly nonlinear variations, are highly effective in modeling parameters that lack a linear correlation, making them suitable for a wide range of applications in predictive microbiology and food safety. For example, RF models have been successfully applied in predicting heavy metal concentrations in Turbot muscle and liver tissues with an accuracy of up to 70% [155].

In many cases, microbial interactions in food involve numerous variables, posing challenges for specifying logistic regression models with optimal biologically relevant forms and interactions [156]. RF models offer a solution by effectively handling complex interactions and large datasets, such as those encountered in food spoilage studies. For instance, Chen et al. [157] utilized RF models to predict quality changes in Pacific white shrimp across a temperature range of −20°–4°C, showcasing their utility in food preservation research. In this study, RF models and Arrhenius models were compared to determine specific markers of Pacific white shrimp quality. The RF models were seen to have lower error rates with values of 0.15%–1.24% (pH), 0.21%–2.82% (thiobarbituric acid), 0.09%–2.51% (total sulfhydryl content), 0.60%–11.84% (Ca^2+^-ATPase activity), and 0.01%–4.76% (total viable counts). The Arrhenius models were shown to have error rates of 0.33%–2.07%, 0.49%–19.84%, 1.73%–23.28%, 2.49%–36.32%, and 11.90%–37.94%, respectively.

Furthermore, RF models have been applied in predicting meat spoilage by integrating metabolome data from various analytical and imaging instruments, demonstrating their versatility in handling diverse types of data [41]. Similarly, Astuti et al. [158] utilized RF models trained on gas sensor array data from fresh chicken meat and *E. coli*-contaminated chicken meat to predict contamination and spoilage based on shelf time, achieving remarkable precision rates of up to 99.25% and 98.42%, respectively.

#### 3.2.2. Support vector Machines (SVMs)

SVMs are highly effective, flexible, holistic machine learning algorithms that can perform linear or nonlinear regression and classification tasks [159, 160]. Support vector regressions (SVRs) are an important branch of SVM applied specifically to regression problems [161]. SVM algorithms are based on a “kernel trick,” a technique that implicitly maps data inputs into high-dimensional spaces and draws a “hyperplane” between them. Basically, it draws a margin between differing data classes. Using the kernel trick technique, the hyperplane is drawn in a way that takes data points as close as possible to it while also maximizing the distance between the margin and the classes, hence minimizing the classification error [162, 163]. The hyperplane is a line (an interface) between two datasets. SVMs can find the most suitable line delineating the two datasets by using linear optimization [163]. Therefore, it can maximize the margin in the high-dimensional space. They are good at handling outliers and are most efficient with well-differentiated data parameters. They are specially used for classification and regression challenges. It is, however, limited by poor effectiveness in handling large and noisy datasets [164, 165]. Despite this, they have been successfully applied in various areas of food safety, including risk assessment in dairy production and prediction of contamination in food samples.

For example, Ma et al. [166] utilized SVMs for risk assessment in dairy production, demonstrating the model's ability to process large datasets efficiently. Bonah et al. [167] employed SVM hyperparameter optimization in combination with data from an electric nose to develop a model capable of predicting *Salmonella typhimurium* contamination in fresh pork samples. Similarly, Zhang et al. [42] trained an SVM model on historical monitoring data to assess and predict the risk during the transportation and refrigeration of strawberries.

#### 3.2.3. Artificial neural Networks

Artificial neural netwoks employs machine learning techniques that simulate the neural pathway of the brain. A basic neural network starts with an input layer of neurons (representing input such as temperature or water activity in a microbiological model), which activates neurons in hidden layers, which then activates neurons in the output layer (depicting predictions from nonlinear activation functions in hidden layers). Each hidden layer contains a network of multiple neurons (also known as nodes or units), the basic unit of computation [168]. The neurons are a logistic regression unit that receives multiple numeric inputs (from other nodes or an external source) and computes one numeric output. The neuron output is computed by a nonlinear function called the activation function. If the weighted sum of the inputs exceeds an internal threshold value within the neuron, the output is activated; otherwise, it is inactivated.

The multilayer model makes it very efficient with nonlinear, complex, high-dimensional relationships between the input and output. However, ANNs require numerical data; hence, they require a transformation of categorical data before they can be used. Deep neural networks, which consist of multiple hidden layers, have been used to model the growth limits of *Bacillus* spp. spores with accuracy greater than 90% [137].

One advantage of ANNs is their parallel architecture, which enables fast computation and evaluation of complex biological processes in food processing operations. They have been successfully applied in modeling diverse food processes such as extraction, drying, fermentation, dairy processing, and quality evaluation. For example, Sun et al. [169] utilized ANNs to monitor the microwave vacuum drying process of carrots, demonstrating their effectiveness in food processing applications.

#### 3.2.4. Gradient Boosting Regression Tree (GBRT)

Models are based on the ensemble learning scheme like the random forest trees [170]. These algorithms work by creating multiple weak models that can be combined into one single model with strong learning capabilities. They are different from conventional boosting algorithms in that the new predictor of GBRT fits the residuals of the previous predictor. GBRT algorithms are designed to reduce loss function by current learner by ensuring that loss function reduces along with gradients direction; that way, final residuals approach zero with continuous iteration, adding up all the tree results to get the final prediction. Like random forests, Gb are tree based; they process interactions effectively, are robust to outliers, and can select variables automatically.

In a study by Sheng et al. [171], GBRT models were trained using multiwavelength data from multichannel infrared spectral sensors and broadband infrared (IR) sensors to predict milk fat and protein content under different conditions accurately. In addition, GBRT models have been developed to predict outbreaks of foodborne diseases by utilizing data from foodborne disease surveillance studies [166].

#### 3.2.5. Logit boost (LB) and Stochastic Gradient Boosting

They are part of a boosting family of algorithms developed in the 1990s [172]. Boosting algorithms function by aggregating (boosting) several weak classifiers (a weak classifier predicts marginally better than random) into an ensemble with improved accuracy [173]. A logit boost model was developed by Benefo et al. [174], making use of whole genome sequence data to identify genes associated with Salmonella stress response in poultry.

### 3.3. Applications of A.I in Predictive Modeling

#### 3.3.1. Artificial Intelligence in Modeling Climatic Factors and Their Effect on Microbial Ecology

Pang et al. [175] utilized logistic regression (LR) and random forest (RF) to analyze the association between weather-related factors and *Listeria* spp. in a mixed produce and dairy farm. Climatic factors, including temperature, precipitation, and wind speed, were considered for analysis. In both LR and RF models, wind speed and precipitation were found to play a significant role in transmitting *Listeria* spp. These experiments demonstrate that both models have good predictive capabilities in analyzing the impact of risk factors, such as weather, on microbial distribution.

Similarly, a random forest-based predictive model was developed by Hwang et al. [176] to quantify the relationship between climatic factors and the presence of *Salmonella* on pastured poultry farms. According to their analysis, the soil model identified humidity as the most significant meteorological variable associated with *Salmonella* prevalence. In contrast, the feces model identified high wind gust speed and average temperature as the most significant. The developed models also showed high accuracy with ROC (area under the curve) values of 0.884 for the soil model and 0.872 for the feces model.

Xu et al. [177] also developed a random forest predictive model that evaluated farm practices and processing variables to identify factors that can reduce the prevalence of *Campylobacter, Salmonella*, and *Listeria* on pastured poultry farms. In the study, the effect of farm practices on the *E. coli* population was used to predict the presence of other foodborne pathogens within pastured poultry farm environment. From the results, the probabilities of identifying Campylobacter in poultry feces and fecal samples at a very low concentration of *E. coli* were 88% and 98%.

#### 3.3.2. Machine Learning Development of Devices for Quality Control

Direct measurement of a product's biochemical quality is cost-ineffective and tedious. As an alternative, time-temperature integration (TTI) has been developed to evaluate food quality by integrating product temperature assessment over time. TTIs are portable devices attached to food products; they assess biochemical interactions in food and then indicate the remaining shelf life. TTIs assess food products for breach of temperature threshold and impact on food quality [178]. These tools are being automated to send warning signs of deterioration indicative of failing storage to food operators.

Currently, AI is integrated into the Internet of Things (IoT) and time-temperature indicators to improve the characterization and detection of cold chain breaches. It assists in defining significant temperature variations, optimizing the placement of sensors (to accurately monitor the load in refrigeration equipment), and developing methods for detecting and characterizing temperature fluctuations.

However, it is crucial to establish well-defined alert thresholds to ensure optimal food safety and minimize wastage. Alerts must strike a balance between being stringent enough to prevent food spoilage and lenient enough to avoid unnecessary disposal of safe food. Achieving this balance requires careful consideration of various factors, including the specific characteristics of the food product, potential health risks associated with spoilage, and logistical costs associated with food waste management [179].

do Nascimento Nunes et al. [180] applied neural networks to predict the temperature of berries in a pallet after training them with ambient temperature data. Subsequently, Badia-Melis. [147] extended this approach to assess fruit temperature dynamics during refrigeration failure using data from cold chain breaks in freezing systems. These two studies demonstrated the effectiveness of artificial neural networks in evaluating the cold chain (in comparison with Kriging algorithms and capacitive heat transfer methods). They also highlighted the effect of sensor number and positioning on results. Results from these studies compared favorably with results from thermal imaging and were also shown to be more effective than methods such as Kriging or capacitive heat transfer.

Other technologies are also being integrated into ML in food quality control. In a case study by Dourou et al. [146], machine learning was combined with Fourier-transform infrared spectroscopy (FTIR) in real-time monitoring of food microbiota during changing storage conditions. The ability of foodborne pathogens (*Salmonella* in this case) to thrive and proliferate during prolonged refrigeration was demonstrated.

#### 3.3.3. Machine Learning in Predictive Modeling of Individual Cell Heterogeneity

A study by Lin et al. [114] compared the effectiveness of four ML models (ANN, RF, GBRT, and SVR) in predicting the single-cell lag time of *Salmonella Enteritidis* after heat and chlorine treatment. The machine learning models were trained with a dataset comprising the following selected variables: population lag times (*λ*), maximum specific growth rate (*μ*max), turbidity detection time (*Td*), sublethal injury rate, and log reduction. It was demonstrated from the study that emerging machine learning models have the potential to predict the single lag time of foodborne pathogens by directly learning from data without assuming underlying mechanisms. The study also demonstrated the importance of population lag time and sublethal injury rate as vital parameters in the single-cell lag time analysis. Increasing population lag time could be positively correlated with a high value of single-cell lag times. The results also provide a framework for food operators to understand the risk from single cells of foodborne pathogens as well as the growth characteristic of *S. Enteritidis* single cells after disinfection treatment [114].

Increases in the mean and variance of single-cell lag time and the population lag time of treated cells showed the effect of external stress on individual cell heterogeneity. Generally, external stress (in this case, from disinfection and heat treatments) is known to play a significant role in increasing individual cell heterogeneity [115]. A comparison of the four models was also made, with ANN shown to have the most accurate prediction of the four models, followed closely by RF. In this study, GBRT and SVR had suboptimal predictions. SVR's unsuitability to handle noise may be responsible for its underperformance [181, 182].

#### 3.3.4. Machine Learning in Predicting Clinical Outcomes from Foodborne Hazards

Machine learning analysis of data from whole genome sequencing of foodborne bacteria pathogens and epidemiological data are being used to characterize food safety risks and predict clinical outcomes. In a study by Njage et al. [131], whole genome sequencing of foodborne Shiga toxigenic *Escherichia coli* was combined with four machine learning models, namely, random forests, support vector machines, and logit boost in hazard characterization and prediction of clinical outcomes from infection. Their results indicated the significance of genotypic data from WGS in determining clinical outcomes by analyzing certain predictor proteins. Genetic predictors of riskier clinical outcomes were shown to include proteins involved in bacterial attachment, genomic islands, sex-pili formation, and protein acetylation. Furthermore, the association between clinical outcome and strain characteristics such as MLST type, lineage-specific polymorphism assay (LSPA), stx-subtype, and sublineage type, and demographic factors such as age and travel were analyzed by ML models. Clinical outcomes were divided into categories ranging from diarrhea, bloody diarrhea, hospitalization, and hemolytic uremic syndrome (HUS).

ML models were trained with a rich dataset comprising sequences from MLST, specific polymorphisms (LSPA-6), and other strain characteristics obtained from STEC outbreaks.

The logit boost model was found to be the best-performing model after comparing its accuracy and similarity with other models.

The application of machine learning helps to overcome the challenge posed by high-dimensional data analysis, which is often required in predicting phenotypic clinical outcomes from microbial genotypes while also considering the intricate interactions between genetic factors that define disease outcomes.

#### 3.3.5. Artificial Intelligence in Pathogen Detection and Growth

Machine learning algorithms have been successfully applied in the detection of pathogens (Table 2). A study by Amado et al. [183] employed a collection of ML algorithms, including random forest, support vector regression, KNN, NBC, and artificial neural networks, to detect the presence of *Escherichia coli* and *Staphylococcus aureus* in beef. Data obtained from emitted gases in meat were used as the input dataset. Results from this study indicated the random forest model to be the most accurate in predicting bacterial contamination. Another study by Tanui et al. [145] utilized the random forest algorithm, the support vector machine, the stochastic gradient boosting algorithm, and the logistic boost algorithm in predicting food sources of listeriosis. The study made use of multi-locus sequence typing (MLST) data of *Listeria monocytogenes* isolates from dairy, fruits and vegetables, meat, poultry, and seafood [145]. Results linked the bulk of infections (32.5%) to fruits, followed by 18.8% in vegetables. This study further highlights the use of genomic data combined with machine learning based on foodborne-disease tracking.  Table 2 shows how various machine learning algorithms have been applied in different fields to predict the risk of highly pathogenic organisms.

### 3.4. Impact of Climate Change on Predictive Microbiology

Climate change generally plays a key role in the burden of foodborne pathogens. At several points along the food chain, changes in weather patterns globally have a significant influence on the emergence of food safety hazards [184]. Elevated water temperatures caused by global warming have been linked with increased expression of virulence genes and faster die off in waterborne bacteria such as pathogenic *Vibrio* spp. [185]. Increased antimicrobial resistance has also been positively correlated with increased local temperature [186]. Globally, changes in microbial ecology due to climate change have been associated with changes in the epidemiology of foodborne infections. A study by Bandyopadhyay et al. [187] showed the association between an increased incidence of diarrhoea and increased temperature in sub-Saharan Africa. Specifically, studies in Ethiopia have demonstrated an increased incidence of diarrhea during the hotter periods of the year [188].

The impact of climate change on changing microbial ecology is readily seen in the dairy industry, where fluctuations in weather patterns are readily associated with changes in microbial ecology. The direct impact of global warming on weather parameters such as temperature and relative humidity plays a major role in increasing the biodiversity of pathogenic microbes in raw milk [189, 190]. Furthermore, warm weather and associated heat stress in milking cows predispose to mastitis and, consequently, higher somatic cell counts and altered physicochemical properties [191]. It is imperative, therefore, that predictive models be developed with consideration of the impact of climatic conditions, seasonal fluctuations in climatic variables, and global warming generally.

A few studies have been carried out on developing predictive models which factor in the impact of climate change. Predictive models that incorporate time-series analysis together with epidemiological data have been used to forecast the significance of environmental temperature in risk assessment of salmonellosis, campylobacteriosis and listeriosis in Belgium [192]. El-Fadel et al. [193] demonstrated the application of climatic forecasts in building Poisson regression models that can measure the correlation between climatic factors and the incidence of morbidities due to water- and foodborne pathogens.

## 4. Conclusion

The review discussed how predictive models are widely used in food microbiology to deduce the growth of microorganisms in food products although these models have some limitations in modeling complex microbial interactions in food dominated by different bacteria populations. The integration of new technologies such as whole genome sequencing (WGS), metagenomics, artificial intelligence, and machine learning and the use of devices based on robotics, the Internet of Things, and time-temperature indicators have, however, been improving the efficiency and accuracy of these models. The integration of machine learning models, in particular, has been greatly beneficial in developing more advanced models. Hence, continued research and development of these technologies are essential for improving food safety and reducing the risk of microbial contamination in food products [194, 195].

## Figures and Tables

**Table 1:** The role of artificial intelligence (AI), whole genome sequencing (WGS), and metagenomics in developing more efficient and accurate predictive models.

Challenge associated with current predictive models	Advances made due to WGS, metagenomics, and artificial intelligence	References
Simplistic, unable to model complex microbial behavior	Metagenomics and WGS provide biological data on complex interactions in food	[108, 109]
	ML and AI are used to visualize and model these interactions	

Errors in measurement and variability	Bayesian modelling and AI approaches provide reasonable estimates of uncertainty and variability	[110–112]
Understanding variability caused by individual cell heterogeneity	Stochastic individual cell modelling based on systems biology and artificial intelligence	[113, 114]

High intraspecific diversity in food	Metagenomic helps in characterising large microbial populations	[114–116]
	AI can be integrated into the development of sophisticated models that can take into consideration intraspecific differences and strain variability	

Insufficient data available on microbial ecology	Whole genome sequencing and metagenomics helps to generate high quality data for predictive models	[117, 118]
	Machine learning and artificial intelligence tools are applied in accurate, high quality data analysis	

Variability among different food materials	Bayesian networks incorporate prior knowledge about the properties of different foods	[37, 119]

**Table 2:** The application of machine learning algorithms.

Application of machine learning	Algorithm	Reference
Risk prediction for highly pathogenic avian influenza at poultry farms in the Republic of Korea	Random forest	[142]
	Gradient boosting machine (GBM)	
	Extreme gradient boosting	

Effect of environmental factors on the abundance of *Vibrio parahaemolyticus* in oyster farms in Taiwan	Extreme gradient boosting	[143]

Prediction of skin flavonoid content from berry physical-mechanical characteristics in wine grapes	Regression trees	[144]
	Random forests	
	Gradient boosting machine	

Food source attribution of *Listeria monocytogenes*	Random forest	[145]
	Support vector machine	
	Stochastic gradient boosting	
	Logit boost	

Microbial quality assessment of chicken liver inoculated or not with *Salmonella* using FTIR spectroscopy	Support vector machine	[146]

Microbial risk assessment using next generation sequencing data in predicting clinical outcomes in Shiga toxigenic *Escherichia coli*	Random forest	[131]
	Support vector machine	
	Logit boost	

Single cell lag time of *Salmonella enteritidis* after heat and chlorine treatment	Gradient boosting regression tree	[114]
	Artificial neural network, random forest	
	Support vector regression	

Temperature prediction in apples	Artificial neural networks	[147]

Predicting perishable food temperatures along the supply chain	Artificial neural networks	[148]

Application of machine learning and RFID in the stability optimization of perishable foods	Kernel logistic regression	[149]

Application of whole genome sequences in determining food sources of HUMAN *Salmonella typhimurium* infection	Logit boost	[150]
	Random forest	

Applying genomic data in determining disease severity in *Salmonella enterica* infection	Logistic regression	[151]
	Random forest	
	Support vector machine, AdaBoost	

Source of campylobacteriosis using whole genome data	K-nearest neighbour algorithm (KNN) eXGBoost	[152]
	Random forest	

Prediction of *Escherichia coli* O157 growth by machine learning	Support vector regression, extremely randomized trees regression	[153]
	Gaussian process regression	

## Data Availability

The data used to support the findings of this study are available from the corresponding author upon reasonable request.
